# Adoption of community-based strategies for sustainable vector control and prevention

**DOI:** 10.1186/s12889-023-16516-8

**Published:** 2023-09-21

**Authors:** Elizabeth Pellecer Rivera, Margarita Rivera Arrivillaga, José G. Juárez, Sandra M. De Urioste-Stone, Elsa Berganza, Pamela Marie Pennington

**Affiliations:** 1 Centro de Estudios en Salud, Universidad del Valle de Guatemala, 18 Av. 11-95 Zona 15 VH III, Guatemala City, Guatemala; 2 Ecology and Environmental Sciences, University of Maine, Orono, ME USA; 3 Present Address: School of Forest Resources, University of Maine, Orono, ME USA; 4 Área de Salud de Jutiapa, Ministerio de Salud Pública y Asistencia Social, Jutiapa, Guatemala

**Keywords:** Diffusion of innovations, Community engagement, Guatemala, Chagas disease, *Triatoma dimidiata*, Vector-borne disease, Neglected tropical disease, Integrated vector control, Environmental risk factors

## Abstract

Community engagement strategies provide tools for sustainable vector-borne disease control. A previous cluster randomized control trial engaged nine intervention communities in seven participatory activities to promote management of the domestic and peri-domestic environment to reduce risk factors for vector-borne Chagas disease. This study aims to assess the adoption of this innovative community-based strategy, which included chickens’ management, indoor cleaning practices, and domestic rodent infestation control, using concepts from the Diffusion of Innovations Theory. We used questionnaires and semi-structured interviews to understand perceptions of knowledge gained, intervention adoption level, innovation attributes, and limiting or facilitating factors for adoption. The analysis process focused on five innovation attributes proposed by the Diffusion of Innovations Theory: relative advantage, compatibility, complexity, trialability, and observability. Rodent management was highly adopted by participants, as it had a relative advantage regarding the use of poison and was compatible with local practices. The higher complexity was reduced by offering several types of trapping systems and having practical workshops allowed trialability. Observability was limited because the traps were indoors, but information and traps were shared with neighbors. Chicken management was not as widely adopted due to the higher complexity of the method, and lower compatibility with local practices. Using the concepts proposed by the Diffusion of Innovations Theory helped us to identify the enablers and constraints in the implementation of the Chagas vector control strategy. Based on this experience, community engagement and intersectoral collaboration improve the acceptance and adoption of novel and integrated strategies to improve the prevention and control of neglected diseases.

**Supplementary Information:**

The online version contains supplementary material available at 10.1186/s12889-023-16516-8.

## Background

Vector control programs are the first line of defense against vector-borne diseases that cause over 700,000 annual deaths globally [1]. With novel intervention tools being developed every year, the need for sustainable and scalable approaches is as critical as the tools being developed. Novel approaches to control tropical diseases have included diverse community engagement strategies to sustain interventions through time [2, 3]. Community engagement (CE), which refers to the process of working together with different local stakeholders [4], has proven to be one of the most important tools to achieve the adoption of health interventions’ goals. Different levels of CE can be attained depending on the objective of the health intervention through a variety of approaches [4]. For instance, the PRECEDE (Predisposing, Reinforcing, and Enabling Causes in Educational Diagnosis and Evaluation) - PROCEED (Policy, Regulatory and Organizational Constructs in Educational and Environmental Development) health planning model, on which we have relied for our intervention, is a tool that facilitates a participatory-based approach that allows to consult and involve community stakeholders as part of intervention planning and implementation efforts to tackle social, biological, and environmental risk factors [5, 6].

An example of this scenario is that despite the growing recognition of the role of social, biological, economic, and political conditions as risk factors linked to Chagas disease, research, and control efforts focusing on these factors have remained scarce [5, 7]. This neglected tropical disease, mainly transmitted by triatomine vectors, continues to affect the most vulnerable populations [8]. In the early 2000s, Guatemala implemented a National Chagas Disease Control Program to prevent vector and blood-borne disease transmission. After a successful campaign using residual pyrethroids for controlling the triatomine vector [9, 10], *Triatoma dimidiata* infestation was reduced up to nine-fold in many municipalities [11]. However, some communities remained with infestation levels beyond the 5% control threshold [9], as the effectiveness of insecticide-based control is restricted by local conditions, sometimes derived from peridomestic habitats [11–14]. This has led efforts to exploring long-term sustainability and continuity of surveillance and control interventions [15].

Between 2010 and 2014, we implemented a pilot program focused on integrated and novel vector control that aimed to improve prevention and control measures through an eco-bio-social approach [5, 6, 16]. This study consisted of a cluster randomized control trial, to reduce vector habitats by addressing the ecological, biological, and social risk factors that facilitate the transmission of Chagas disease in Comapa, a region with persistent triatomine infestation [5, 6, 17]. This integrated approach allowed us to define community, intersectoral, and participatory management activities within the ecosystem. By analyzing potential risk factors associated with *T. dimidiata* domiciliary infestation, as well as *Trypanosoma cruzi* reservoirs, we identified that the most relevant risk factors were dog density, mouse presence, interior wall plaster condition, dirt floor, tile roofing, and coffee tree presence [5]. To tackle the risk factors identified we developed a community-based intervention that consisted of modified insecticide spraying, an educational process regarding Chagas diseases and the risk factors identified, and participatory rodent control. The post-test evaluation showed a significant increase in knowledge levels regarding Chagas disease and prevention practices in the intervention communities. Additionally, control communities showed higher odds of nymph infection and rat infestation (8.3 and 1.0-fold, respectively) than infestation communities [6].

Herein we present an insight into the process of adoption of the intervention by the participant communities, obtained by an interim evaluation. To understand the adoption of the proposed intervention, we use concepts from the Diffusion of Innovation Theory (DOI) [18]. The DOI analyzes the processes of how an innovation spreads and is adopted by a population, depending on certain characteristics of both the innovation itself and the target population [18]. We focus on the attributes of each of the activities proposed (“innovations”) to understand its influence on the level of acceptance by study participants. Our objective is to better understand community engagement practices and innovation characteristics that enabled or hindered the adoption of vector and parasite reservoir control interventions. Our findings provide insights regarding community engagement for local vector control programs to improve their sustainability and acceptance in other regions, and innovation characteristics that need to be considered when planning community-based health interventions in rural settings.

## Main text

### Study site

**Fig. 1:**
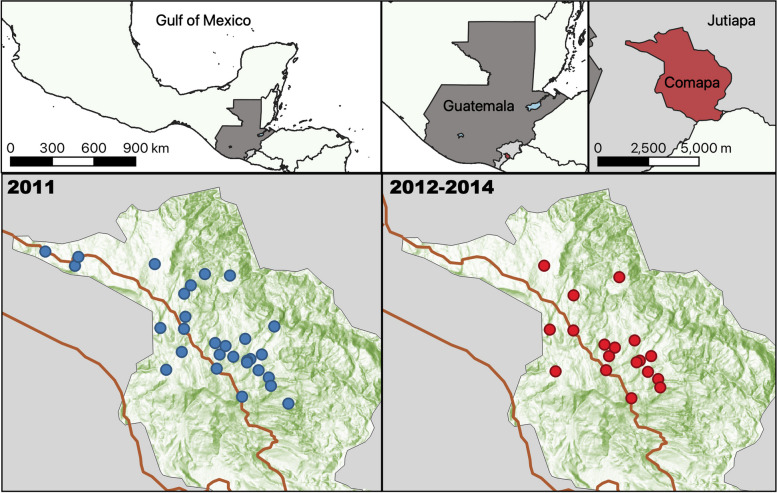
Location of Comapa and participant communities. Blue circles show the location of communities involved in the situational analysis of 2010-2011, used to identify eco-bio-social risk factors [5]. Red circles show the location of the communities selected to be part of a Cluster Randomized Control Trial from 2012 to 2014. “Created with BioRender.com”. Map generated using QGis 3.18 using freely available administrative boundaries and ESRI World Hillshade. (Source: http://services.arcgisonline.com/arcgis/rest/services/Elevation/World_Hillshade/MapServer)

The municipality of Comapa is located in the department of Jutiapa, Guatemala (Fig. 1), and borders El Salvador. Comapa is mostly rural with a population of 32,000 inhabitants, the two largest ethnic groups are Ladino (87%) and Xinca (11%). A quarter of the population lives in poverty, and the main economic activity is agriculture, maize and beans as the staple crops. In 2018, only 22% of the school-age population reached secondary education, while 25% reported no formal education [19]. Different governmental and non-governmental institutions promote comprehensive development locally [20]. Comapa maintained triatomine domiciliary infestations above 25% after multiple insecticide applications [5], and it has been a region with persistent triatomine infestation and transmission [17].

### An integrated community-based eco-bio-social vector control intervention

**Fig. 2:**
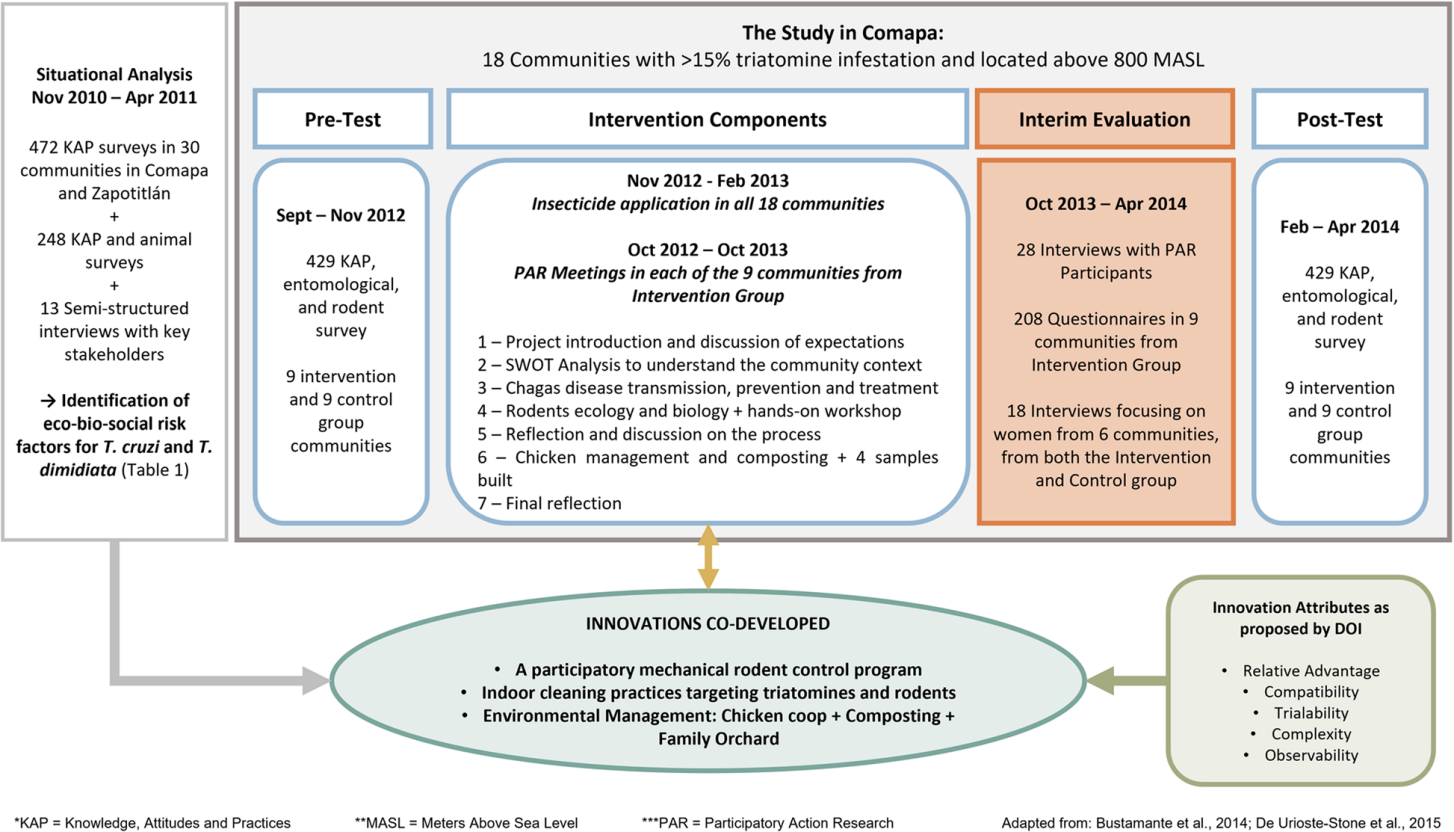
Description of the stages, data collection methods, participants, and activities performed in the overall project. This manuscript is solely focusing on reporting the results of the interim evaluation, which aimed to understand the adoption process of the intervention

The study consisted of a cluster randomized pre-test post-test experimental design, combined with a participatory action research (PAR) intervention (Fig. 2). The aim was to tackle the risk factors associated with the infestation of triatomines in the household, which were identified in the situational analysis, through KAP (knowledge, attitudes, and practices), entomological (*Triatoma dimidiata*), and animal (chicken, rodents, and dogs) surveys (Table 1). The PRECEDE-PROCEED health planning model guided the development, implementation, and assessment of the study [5, 6]. Eighteen communities with high infestation levels (above 15%) were selected for the study and were randomly assigned as control and intervention groups. Within each community, a cluster of 24 households was selected, using a probability systematic sampling design; with the exception of one community that had only 21 households, thus all the households were included [6]. During the visit for the pre-test surveys, participants were given brief notice about the series of meetings that were to take place within the following months.

The intervention consisted of a modified application of insecticides and a series of seven educational meetings with participants (9 intervention communities, 24 houses per community). The topics of the PAR meetings addressed each of the risk factors previously identified for the region during the situational analysis (Table 1), and were shaped during the PAR process through consultation with the participants [16]. The activities in the meetings were based on interpersonal communication, with the members of our team taking the lead, providing the corresponding information, and using questions to create a two-way communication. In each meeting we used supporting material, like flipcharts, puppets, music, videos, and games, aiming to engage participants. The meetings took place in each of the intervention communities, in different venues proposed and offered by the participants (i.e., the school, someone’s house, the community room).

**Table 1:** Risk factors identified and proposed innovations to mitigate them [5, 6]

Risk factors defined by baseline surveys	Proposed innovation	Definition
26% (16–39) rodents infected with *T. cruzi* Association of triatomine infestation with rodent presence OR 4·0 (1·1–14·1)	A participatory mechanical rodent control program.	Participants were provided with one cage and two different-sized snap traps. Meetings were conducted to discuss rodent ecology and biology, including workshops for training and practicing trap use, cleaning, and safety measures for handling and sacrificing rodents once they were captured.
Socioeconomic factors associated with triatomine infestation: earthen floors OR 3·4 (1·9–6·0) bajareque walls OR 1·9 (1·2–3·9) tile roofs OR 1·9 (1·1–3·3)	Indoor cleaning practices targeting triatomines and rodents.	Meetings about rodent ecology were complemented by recommendations targeting potential food sources for rodents and potential habitats for rodents and triatomines. For maize, a staple food grown in the area, recommendations included elevating and moving stored grain away from walls to prevent rodent nests, and removing all grain spilled below the metal silos to reduce food sources. Triatomine reduction strategy included periodic surveillance of potential sites for hiding and living (i.e., behind posters and frames on the wall, chicken coops, etc.).
Ecological and environmental factors associated with triatomine infestation: coffee trees OR 1·9 (1·2–3·2), jocote trees OR 1·8 (1·1–3·2), hens roosting inside OR 2·0 (1·2–3·3)	Environmental Management: Chicken coop + Composting + Family Orchard.	Composting methods were promoted to reduce leaf litter and organic waste to limit potential rodent food and nesting sites. The use of a chicken coop was proposed to have a safe space outside the household, paired with composting as a source of food for poultry. Four sample chicken coops were built in households that showed high participation and previous triatomine infestation. We collaborated with an NGO and the Ministry of Agriculture and Range to provide seeds, so the composting could be used in a family orchard.

From the seven meetings, we held three directly targeting the risk factors: one introducing Chagas disease, one regarding rodents’ ecology and biology for its control, and one regarding chicken management and composting (Table 1). The rodents’ meetings proposed the use of traps for rodent control and was accompanied by a practical workshop. We set the traps in a volunteer house and picked them up the following day, aiming to demonstrate the traps use, and the killing and handling of rodent process, which was usually done by a volunteer. For environmental management, we promoted the use of chicken coops with an integrated compost. These meetings also included a practical activity, where we built four sample chicken coops in four different communities, aiming to serve as an example for participants and neighbors.

**Fig. 3:**
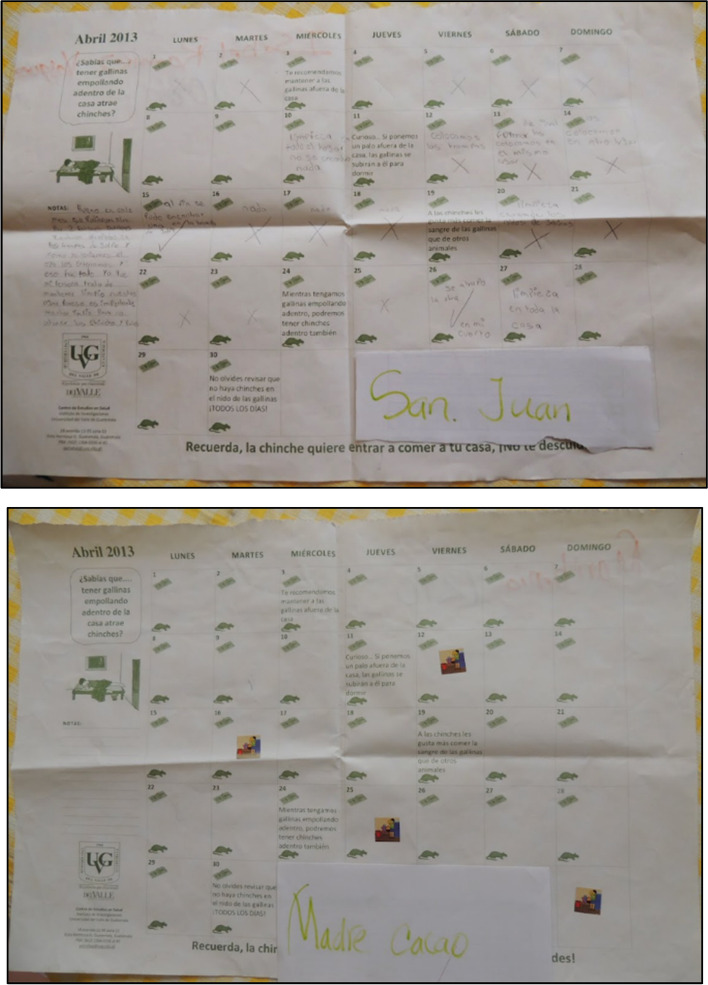
Examples of the calendars that were used as a tool for the participants to track the activities they were adopting

To record and follow up on how the activities suggested in the PAR meetings were being carried out, we provided participants with a calendar where they could write and report what they were implementing (Fig. 3) [6, 16]. This helped us reinforce the comprehensive approach and community engagement for the proposed innovations. The other four meetings had a reflective approach, one presenting the project and discussing expectations, one including a SWOT (Strengths, Weaknesses, Opportunities, Threats) analysis at the beginning of the process; and two reflective meetings halfway through and at the end of the process to promote active dialogue and reflection among participants [6]. These four activities aimed to offer a space for exchange, to understand participants’ expectations, get their input, and co-define the details regarding the innovation strategies.

### The intervention as an Innovation

Based on the situational analysis, we identified three major activities addressing risk factors as innovations (Table 1). We focused on preventive innovations, as high adoption of the intervention should decrease the possibility of unwanted future events, in this case, the transmission of Chagas disease [18]. Each innovation had its own characteristics (“attributes”), which could influence its rate of adoption [18] (Table 2). We conducted an interim evaluation after the implementation of the intervention, to analyze participants’ perceptions of the five innovation attributes proposed by DOI [18], to identify factors that hindered or facilitated the adoption of the intervention components. Previous studies have found that innovation attributes serve as predictors of adoption and diffusion intentions in the population [21, 22], and are related to the sustainability of interventions [23].

**Table 2:** Attributes of an innovation from the diffusion of innovations theory [18]

Attribute and definition	Examples, as applied to the innovations proposed
**Relative advantage**: Perception that a proposed innovation is better than the idea or method used previously.	a. Poison vs. mechanical rodent traps: The rodent traps were considered better than using poison (the most widely used method previously). Some of the benefits identified were that individuals had more control over the rodent carcass, and the poison had an unpleasant smell. b. Routine indoor cleaning vs. inclusion of practices targeting triatomines and rodents: The benefit of moving the things around the house when cleaning allowed individuals to find triatomines and identify rodent nests. c. Chicken coop vs. free-range chicken / Not having compost vs. composting organic waste / Not having an orchard vs. having a family orchard: Creating a compost within the chicken coop served as a food source for the chickens, so they did not need to roam around, and provided more control from individuals over their animals. Having an orchard allowed the individuals to produce vegetables for family consumption.
**Compatibility**: Perception that a proposed innovation is coherent and adapts to the values, experiences, and needs of the people to whom it is addressed.	The innovations proposed did not always seem to be perceived as directly related to triatomine control, but to tackle people’s other current problems (i.e., rodents), making the innovations compatible with the context and needs. Most participants were farmers, so they had previous experience with chickens and agriculture. Participants mentioned that they were willing to share their innovation with neighbors, or that they believed other people in the community would adopt the innovation, if available, showing a perceived acceptance within a larger context and its values.
**Complexity**: Perception that an innovation is difficult to understand and use by the people to whom it is addressed.	Participants reported that using traps was easy (some preferring one trap design over the other) and were being used by a diversity of users (children, adults, women, men). Age or health conditions constrained participants to implement cleaning activities that required moving furniture or other heavy items. Some people found economic difficulty to adopt the innovations when they were required to purchase materials to be able to implement them.
**Trialability**: Degree to which an innovation can be tested or experimented with before its full adoption.	The workshop held was found useful to try and learn how to use the traps. Participants had previous experiences with chicken coops and family orchards, whether from their own initiative or promoted by other projects.
**Observability**: Degree to which an innovation results or outcomes are visible to others.	Traps showed immediate results in controlling the rodents in the households. Neighbors and family members could see the results of using rodent traps, and some borrowed traps from the participants. Some participants could not see the results of the orchards, because the weather damaged the crops. People not involved in the project wanted to participate after looking at their neighbors’ results, or after talking with the neighbors about the benefits and learning experience.

### Data collection

We conducted an interim evaluation using mixed methods, mainly targeting the participants from the 9 communities in the Intervention Group (Fig. 2). We used questionnaires to assess the adoption rate, and semi-structured interviews to gain a deeper understanding of perceptions regarding the intervention itself, the adoption processes, and the practices. Both instruments addressed perceptions of knowledge gained via the education component of the intervention, the level of adoption of the intervention, perceived attributes of the innovation, and factors that facilitated and limited the adoption of practices.

We conducted a total of 208 questionnaires during an interim evaluation between October 2013 and March 2014. These questionnaires targeted participants from the 24 households in each of the 9 communities who had been previously allocated to the intervention group. Although in the pretest there were 216 households included, eight did not participate in the interim evaluation due to reluctance or absence. Additionally, we held 28 individual or group semi-structured interviews with 31 community participants. We used purposive sampling, using a maximum variation strategy to capture individual rich descriptions while seeking a wider range of perspectives [24]. We considered the following conditions: range of communities (all nine intervention communities); age groups (child, young adults, middle-aged, and seniors); gender (female and male); and different levels of participation (high, medium, low) in the intervention meetings.

We also conducted 18 additional interviews in 2014 focusing on women as change agents, given that most of the participants in the PAR process were female. This set of interviews were conducted with the aim of comparing the perspectives between women from the control and the intervention communities [20]. We used a maximum variation sampling strategy [24] based on age, occupation, and marital status. To compare experiences, this set of interviews included participants from 6 communities, half from the intervention group and half from the control group. For the intervention group, we focused on women with higher attendance to PAR meetings ( > = 5) [20].

### Data analysis

We conducted descriptive analyses for the survey responses using both SPSS® and STATA17®. We coded and analyzed data from the interviews using NVivo12®, using pseudonyms to ensure confidentiality. We recorded and transcribed verbatim most interviews to capture the essence of meanings as portrayed by participants [24]. For the qualitative analysis, we used a deductive approach for the first cycle coding, using the DOI five attributes as our conceptual framework. For pattern coding we utilized the DOI theory, as applied to our research questions, to understand the adoption process [25].

### Quality assurance

Overall, we used three validation strategies: prolonged engagement in the field during data collection; triangulation across sources and methods during data analysis; and peer debriefing between Universidad del Valle de Guatemala (UVG) researchers and Ministry of Health (MoH) collaborators to enhance the interpretation of results [25, 26]. Additionally, we used the COREQ (COnsolidated criteria for REporting Qualitative research) Checklist to provide more detail of the study design and data collection process (Additional File 1) [27]. Although this checklist was designed to report the essential items of qualitative research, we also included information about the questionnaire process when relevant and applicable.

## Results

### Participation in the PAR process

We started the PAR process with 216 participants, representing the households selected from the 9 intervention communities, and after a year 10 participants withdrew. Additionally, 22 people who were not part of the original intervention group, joined the project officially due to their active and consistent participation to the activities and meetings organized ( > = 3). Attendance levels per meeting varied, 44% (96/216) of the households had high participation (6–7 meetings), and only 13% had low participation (0–2 meetings) [6]. Most attendees were women (~ 78%). Several study participants that played key roles in their communities or that participated regularly in initiatives from other institutions (i.e., NGOs, churches, municipal offices) attended meetings regularly. This inherent leadership was also shown during our intervention, as they often had higher attendance, active participation, or volunteered to support the organization of our monthly meetings.

The main barriers that interviewees identified to attending the PAR meetings were health- or family-related responsibilities (i.e., doctor or school appointments, ill relatives, or because they were not informed about the activities), and men’s absences because of agricultural work. The primary motivations to participate mentioned were the possibility to tackle multiple problems (i.e., insecticide spraying for triatomines, learning new information), and to address rodent issues. In some cases, younger sons or daughters of the participants would attend representing their households.

“*I said yes [to the project] because we wanted to see the house clean, that there were no animals [kissing bugs]. Those animals are very bad because they bite you.*” (Dolores, 2013, female, 32 years old).

“*What I liked the most was the way they came to teach us how to get rid of mice, how to get rid of kissing bugs.*” (Manuela, 2013, female, 43 years old).

### Participatory mechanical rodent control program

According to survey results, the most adopted innovation was the use of rodent traps as 98% (*n* = 203/208) of intervention participants reported using them in the previous year. Most people (35%, *n* = 70/203) reported using the traps one or many times a week (Table 3). From 203 cases where people had used a rodent trap at some point in the year, 20% (*n* = 41/203) killed the trapped animals. Most people, 84% (*n* = 171/203), reported burying the animal, as shown during the training for safety purposes.

**Table 3:** Adoption, compatibility, and observability attributes reported regarding the use of participatory mechanical rodent control

Innovation Attribute	Responses	No. positive responses/ total (%)
Adoption	Use traps the previous year	203/208 (98)
Compatibility	Willing to buy new rodent traps if theirs ruin	145/203 (71)
	Think other people in the community would be willing to use traps, if accessible	136/203 (67)
Observability	Willing to lend their traps to neighbors	132/203 (65)

Participants identified a **relative advantage** of using traps when compared to the previous methods used (i.e., poison), most reported having stopped using the previous method. Participants reported that traps allow them to have more control over the disposal of rodent carcasses, as poisoned rodents tended to die in their burrows where it was difficult to track and dispose. Additionally, participants acknowledged that traps represented a lower risk for human and animal health in the households (i.e., animals getting poisoned). In the mid-term, traps represented a cheaper option than purchasing poison regularly.

“*We killed them like that [with poison], but it was bad because they died inside the holes we had in the walls, and it was difficult to get them out. They bothered there; they stank. On the other hand, with the traps it is not difficult because they remain there, then we take them out and throw them away*.” (Dolores, 2013, female, 32 years old).

“*What has helped me the most is that I no longer want mice in the house because they say that kissing bugs also depend on mice*.” (Sonia, 2013, female, 32 years old).

Rodents were already perceived as annoying, and a threat to people’s grain storage, food, and personal items, which facilitated the **compatibility** of this innovation to tackle a current problem in the local context. The traps made participants feel empowered to control rodent presence. Participants showed interest in using traps in the mid-and long- term; the survey showed that 71% (*n* = 145/203) of the intervention participants would be willing to buy a new rodent trap if theirs got lost or broken. Most of the participants thought of traps as an innovation that the rest of the community would be interested in, as 67% (*n* = 136/203) thought that other people in the community would be willing to use rodent traps if they became accessible (Table 3).

Regarding the **complexity**, most interviewees considered that it was easy to learn (21/27) and to use (20/27) the rodent traps. We decided to offer two types of traps, slam traps and cages, as we found mixed opinions during the PAR process. Slam traps were found easy to use because they killed the rodent immediately, but more users were afraid of hurting themselves. Cage traps were identified as easier to set up but posed the challenge of handling a live rodent. Both types of traps were reported to be used by children (from 10 years old) and adults (up to 80 years old). Only 9% (*n* = 19/203) of surveyed people did not use the traps. Of the listed reasons why they decided not to use the rodent traps, none seem to be related to the complexity of using it (i.e., not having sight of rats or mice, having lent the trap to someone else and not having it gotten back, etc.). Some of the general difficulties mentioned in the women’s interviews also referred to the installation of traps in high places, the fear of killing the trapped mice, or that some rodents were too big to fit in the trap (although a participant shared the creation of their trap).

During the PAR meetings demonstrative workshops and follow-up activities, participants could try using the traps and ask questions, providing a **trialability** opportunity. Most of the interviewees (24/27) reported that the demonstration workshop was very helpful in learning how to use rodent traps. The **observability** attribute of this innovation was challenging. Since traps were used at night, most interviewees reported that they could not observe how their neighbors used them. However, 65% (*n *= 132/203) of survey respondents were willing to lend their rodent traps to neighbors (Table 3).

*“I have caught a lot of mice. Last night my husband was telling me to set the traps again because the mice are back in here again.”* (Dolores, 2013, female, 32 years old).

“*I told all the people I invited them to the meetings; we told them about the benefits of the traps. Only one person went to the meetings because I lent her a trap for a week and there were too many mice. The person came and caught quite a few. Now only sometimes they ask me what the meetings are about or what I am going to learn there, but there are people who do not pay attention.*” (Irma, 2013, female, 36 years old).

### Indoor cleaning practices targeting triatomines and rodents

This innovation encouraged the adoption of in-depth cleaning practices targeting rodents and triatomines, but each participant adopted what was more appropriate for their household. During the meetings we suggested some cleaning activities, prioritizing tackling rodents and triatomines. Survey results showed that the activities participants most often adopted were sweeping the floor (84%, *n* = 175/208), moving things around regularly (53%, *n* = 111/208), and sweeping or cleaning the walls (44%, *n* = 92/208) (Table 4). Other practices like covering food containers, picking up grains, cleaning behind the corners, or behind the furniture and frames were adopted by only one-third or less of the participants.

**Table 4:** Adoption and complexity attributes reported regarding the use of indoor cleaning practices targeting triatomines and rodents

Activities proposed	No. positive responses/ total (%)			
	**Adoption**	**Complexity**		
		**Easy**	**Regular**	**Difficult**
Sweep the floor	175/208 (84)	148/175 (85)	20/175 (11)	7/175 (4)
Move things around regularly	111/208 (53)	67/111 (60)	17/111 (15)	27/111 (24)
Sweep or clean the walls	92/208 (44)	74/92 (80)	13/92 (14)	5/92 (5)
Sweep the nooks, corners, and behind the things	68/208 (33)	55/68 (81)	9/68 (13)	4/68 (6)
Clean behind the frames	64/208 (31)	52/64 (81)	5/64 (8)	7/64 (11)
Cover the food containers	46/208 (22)	38/46 (83)	4/46 (9)	3/46 (7)
Pick up the grains from the floor	23/208 (11)	18/23 (78)	2/23 (9)	2/23 (9)

The major difference between the proposed innovation and the previous practices lies in the promotion of deep cleaning practices which included moving things around, checking or cleaning the walls, and cleaning around the house. As the practices adopted varied, the **relative advantage** reported by some of the participants was that they were able to notice burrows or rodent tracks and find triatomines or other insects.

“*What helped me the most was cleaning well inside the house so that kissing bugs and mice don’t find a breeding ground. That’s what I like the most because sometimes you put things in a corner and don’t sweep well, that’s where they find a place to breed.”* (Martina, 2013, female, 31 years old).

The risk factors identified were related to socioeconomic conditions, however, aiming for **compatibility** we focused on cleaning practices instead of house improvement (i.e., plastering walls, etc.). Participants reported having added new activities to their usual cleaning. Reported practices included taking more time to move furniture, boxes, beds, and other things that were close to the walls. They also paid more attention to cleaning around the house (patio), having food covered, and picking up grains that might had been left on the floor. Each participant adapted the proposed practices according to their context and capabilities, reporting that although they had put more effort in, the novel cleaning practices were not performed daily.

Regarding the **complexity**, the activities’ level of difficulty varied and was related to having to spend additional time and/or effort than usual. Most of the respondents considered easy the three most adopted activities (85%, *n* = 148/175 sweeping the floor; 60%, *n* = 67/111 moving things around regularly; 80%, *n* = 74/92 sweeping or cleaning the walls). Moving things around regularly was also the activity considered most difficult by the people that were implementing it (24%, *n* = 27/111), but it was the second activity most adopted (Table 4). In general, women were in charge of cleaning the houses, and in the case of older women, sometimes they required the support of men or other members of the household to move things, like heavy furniture, to clean.

“*Before, I had people to help me in the house to do the chores and now I don’t. It’s up to me alone to be working here at home.*” (Laura, 2013, female, 50 years old).

There was no space for the **trialability** of these innovations during the participatory process, but the interviews showed that the innovations were adopted according to the needs and possibilities of each household. The **observability** attribute was challenging. Interviewees reported that they were not aware of their neighbors’ housekeeping practices, although in the meetings we suggested some specific activities. However, they mentioned that they observed more cleanliness and order in their own home, which was perceived as a more pleasant and healthy environment.

### Environmental management

The innovation consisted of three different but complementary strategies: composting, building or using a chicken coop, and a family orchard. This was the least adopted innovation, as it required certain conditions (i.e., owning chickens, having outdoor space for the coop and orchard). Survey results showed that 45% (*n* = 93/208) of intervention participants had a compost, 48% (*n* = 100/208) created an orchard, and 46% (*n* = 95/208) built a chicken coop (Table 5).

**Table 5:** Adoption and complexity attributes reported regarding the implementation of environmental management strategies

Activities proposed	No. positive responses/ total (%)			
	**Adoption**	**Complexity**		
		**Easy**	**Regular**	**Difficult**
Compost				
Created a compost during the previous year	93/208 (45)	36/93 (39)	22/93 (24)	33/93 (35)
Still maintain the compost / Compost maintenance	48/93 (52)	51/93 (55)	19/93 (20)	20/93 (22)
Chicken coop				
Created a chicken coop	95/208 (46)	51/95 (54)	15/95 (16)	28/95 (29)
Still maintain the chicken coop / Chicken coop maintenance	85/95 (89)	61/95 (64)	21/95 (22)	12/95 (13)
Family orchard				
Created the family orchard	100/208 (48)	46/100 (46)	26/100 (26)	27/100 (27)
Still maintain the family orchard / Orchard maintenance	28/100 (28)	41/100 (41)	28/100 (28)	30/100 (30)

We found mixed results regarding the **relative advantage** of these innovations. The most common practice in the area was to let chickens out during most of the year but to keep them caged or indoors during the sowing period. Interviews showed that some people put the compost inside the chicken coop and experienced the benefit of chickens getting fed by the compost. However, some people perceived that the behavior of the confined hens changed in an unproductive way, so they preferred to let them loose. Regarding the family orchard innovation, we found plenty of interest in receiving donated seeds as the participants recognized the economic benefits of growing their own vegetables.

“*One day I killed one [chicken] and I told my husband: “How fat is this hen that was locked up”, He told me: “That happened because the compost is already there and it [the chicken] is eating little animals*”.” (Dolores, 2013, female, 32 years old).

The three activities proposed had **compatibility** with the local context. Some participants had worked with organic compost and chicken coop as separate projects, but not everyone had continued. The greatest motivation to start the orchard was to take advantage of the seeds that were donated. Interviews suggested that few people had chickens, hence the lack of interest in the coop. Most people in the locality worked in agriculture, however, the seeds provided for the orchard were different from traditional crops.

“*I had the seeds, I had to sow them*.” (Roberta, 2013, female, 30 years old).

These innovations were perceived as the most difficult ones among what was proposed. **Complexity** was due to the fact that for its implementation there was a need to have space, buy the necessary materials, and invest time in construction and maintenance. Only 28% (*n* = 28/100) of them still had an orchard at the time the questionnaire was conducted (Table 5). Some of the reasons to abandon the orchard were lack of seeds, water, or time availability. About 48% (*n* = 45/93) of participants had already stopped composting before the survey was conducted. Other reasons that interrupted having an orchard included plagues or animals that had eaten the garden (roosters, chickens, and cows). Some of the factors listed as reasons to abandon the composting were not having enough water, organic material, space, or time. Other reasons listed were having it been destroyed by animals (pigs, chickens, cows) or that the project had come to an end, so the support stopped as well.

“*Sometimes the soil is hard, when you have this organic fertilizer that you take from the compost bin, you can mix it and with that you get a softer and more nutritious soil so that the plant grows well, although now it hasn’t grown much because sometimes too much water doesn’t let anything grow*.” (Rafael, 2013, male, 50 years old).

For the **trialability** attribute of this innovation, we relied on the four pilot chicken coops in the intervention﻿﻿ communities, since some participants already had chicken coops at home, as well as a structure for the garden and the compost bin. Many of the participants affirmed that our intervention triggered their will to implement one or more activities in their houses. Given that these innovations were located outside the houses, there was some opportunity for **observability** among neighbors. However, participants reported that they did not know about their neighbors’ chicken and outdoor management practices.

“*I used to have them [the chickens] all over the house and now I keep them in one place. I vaccinate them and take care of them more.*” (Laura, 2013, female, 50 years old).

## Discussion

The results showed a diverse rate of adoption of the innovations proposed. We used the DOI attributes for analysis, as they have shown to be predictors of adoption in diverse topics and could be used in the design of future interventions [21, 22, 28–30]. However, the adoption process does not happen in a vacuum and can be influenced by external factors like social norms, attitudes towards innovations, knowledge, and channels of communication [18, 21, 22, 31]. Using a qualitative approach allowed us to better understand the factors that enabled and hindered the process of adoption, beyond the innovation attributes. Using the SWOT analysis and reflective approach in the intervention process helped us engage and jointly develop the intervention activities with the communities while tackling the risk factors identified. Throughout the intervention process, community engagement served as a transversal enabler of implementation and adoption.

### Attributes and adoption of the innovations

The attributes that seemed to play a key role in the adoption of our intervention were complexity, compatibility, and relative advantage. Trialability and observability attributes showed mixed results, only being important for the adoption of rodent traps. Prior studies have shown that innovations can be rejected if they are perceived as too complex to understand and use [23]. Perceived simplicity and innovations easy to use have been related to the higher acceptability [29] and adoption [21]. Rodent traps were the most adopted innovation and participants showed consensus that both traps were easy to use. In contrast, we found mixed results in the difficulty perceived for indoor cleaning and environmental management, as they demanded more physical effort and resources investment, and could be influenced by external factors (i.e., weather). The fact that the project provided traps to the participants served to mitigate the complexity, as for environmental management the investment of economic resources was a strong limitation. It is fundamental to understand the socioeconomic context where the innovations are proposed, to foresee and mitigate external factors that can constrain adoption, beyond individual will.

The innovations proposed were compatible with the local context, values, and needs of the participants. Compatibility was mentioned in all the innovations, not only by being appropriate to the context and practices but also suitable to solve current tangible problems. The incompatibility of innovations can obstruct adoption partially or completely, despite the knowledge level that adopters might have [31–33]. For example, in the case of the rodent traps, even if our objective was focused on halting Chagas disease transmission, for most of the participants’ rodents’ presence in their houses was a more tangible problem and the traps became an instant solution.

Along with that, the concrete results provided by rodent traps were immediate proof of its relative advantage. Rodent traps were associated with individuals’ empowerment, as having traps increased their perception of control and efficacy over the issue in a short period. Although relative advantage has been found in other studies as one of the best predictors of adoption [18, 33, 34], we obtained mixed results, as for the indoor cleaning and environmental management innovations, it did not seem to be the most important attribute by itself [21, 29]. Additionally, demonstrating the benefits of preventive innovations is sometimes challenging, as the outcomes are not seen immediately but at some point in the future [18].

Although the observability attribute had a more limited application in our project, other studies have shown that when innovations have visible benefits, users tend to adopt [21, 23, 34] and maintain them [23]. In this case, rodent traps had high visibility, as participants could see an immediate benefit. The reported traps’ sharing with neighbors and family members showed a ripple effect in the communities and that the innovation was visible beyond participants. On the opposite side, the benefits that can be obtained from the orchard, chicken coop, and compost take a long time. Consistent with the literature [23, 29], these innovations had lower rates of adoption. Trialability has been reported as a factor that facilitates users to adopt and maintain innovations [18, 21], and lack of it can obstruct the diffusion process [32]. The trialability activity held through the practical workshop was key to facilitating the adoption of rodent traps, as most of the participants learned there how to use them and how to manage the rodent. Although we held a few trialability activities to show the environmental management innovations, the adoption was constrained by other factors.

Overall, one of the largest barriers to adoption was economic resource availability. Given that we were working in a rural context, where most people had a lower income, even if participants were willing to try the innovations the constraint was that they could not purchase the materials or tools needed. Knowledge regarding Chagas disease transmission and practices regarding rodent control and chicken management changed after the intervention [6]. Environmental management practices are sustainable over time, participants associated the innovations with strategies for a more general well-being and healthy household. However, a barrier to evaluating long-term adoption was that the follow-up process was limited by the resources of the project, which depended on the funding timeframe.

### Community engagement as an enabler of the intervention and the challenges we found

The project considered community engagement at the base of the participatory strategy. The fact that the intervention was developed in consultation with the community, taking the context into account, was essential, as the ideas were shaped by the participants’ input. One of the key components of the process was rapport and trust building with the intervention participants and stakeholders [6, 16]. As a community-based project, participants were involved in different levels along its stages [4]. The PRECEDE-PROCEED Model allowed us to work together with the community before and during the PAR meetings, informing and consulting with community members to link the risk factors identified with local problems and practices that they felt the need to change [4, 5]. This had an impact on each of the DOI attributes as the changes proposed by the project had been designed to respond to context-specific factors and enabled the adoption of each innovation.

The random sampling of our study showed that some of the intervention participants simultaneously played key roles in their communities or regularly participated in the development of projects and initiatives of other institutions (i.e., NGOs, churches, municipal offices). Although we had no control over this aspect of participant selection, this condition led to an advantage in terms of the adoption of the innovations. People had a certain habit of attending meetings, following up on interventions, and playing a leadership role with their neighbors (i.e., they talked with their neighbors and other family members, and were more willing to adopt and participate). Within the DOI Theory, opinion leaders play a role in influencing the diffusion of innovation processes [28, 30]. However, the random sampling also limited the participation of others who were interested in the intervention.

In this project, community members’ participation was essential, as they represented the main group with whom the most community engagement was generated. However, development agencies and municipal institutions in Comapa regularly work with local actors who play different roles in their communities. For our interventions related to environmental management, we coordinated with two institutions that worked on issues of agriculture and child nutrition. These local intersectoral coalitions allowed us to identify potential local partnerships to implement our project [4].

### Sustainability

Although we proposed the intervention as an integral strategy for Chagas disease vector control, it was useful to break it down and assess each of the activities, as each had different enablers and challenges. This strategy has been useful to evaluate sustainability at a finer level than approaching a program or intervention as a whole [23]. Innovations attributes from DOI theory are useful characteristics to take into account when designing an intervention [22, 23].

Since Comapa is one of the regions with persistent Chagas disease transmission, there are other institutions, including the MoH, working on the topic since decades ago. Our project benefited from these processes already taking place, including the rapport and collaboration with local institutions. Although we obtained satisfactory results with the implementation of the project [5, 6, 16], it is common to have sustainability challenges after the funds end [23]. Institutionalizing integrated vector control strategies could strengthen current efforts and the sustainability of novel interventions, as it would assure the allocation of funds and resources within the national framework. However, taking that step often takes time, resources, and political will. In a most immediate manner, and especially with activities that can be implemented by the individuals and the community itself, we believe that implementing different strategies of community engagement serves to facilitate the adoption process and to sustain the activities through time. Although this experience was focused at the community level, horizontal approaches could also benefit institutional-level interventions through intersectoral coalitions and partnerships.

### Limitations

One of the initial challenges in the study was the design itself, combining a randomized control trial with the PAR methodology. The random probabilistic selection allowed us to have a diverse group of participants, which we consider a strength of the study. However, since the initial home visits and the meetings took place during weekdays and daytime (our working hours) we had an overall higher participation of women, as they were the ones available at that time. This is not necessarily a negative aspect, but we believe it created a gender bias, by leaving men out. In addition, although the objective was to be able to compare the control and intervention groups, we acknowledge that there could have been external factors (personal, social, political) outside the scope of the project, that could have influenced the results obtained (i.e., spillover information between communities) and the participation of the people involved (i.e., political disagreements between participants).

In this study, we are mainly presenting the perspective of participants from the intervention group. It would be ideal to conduct a similar study in a mid- and/or long-term period, being able to include longitudinal data on the adoption of innovations, and the experiences from people in neighboring communities, to better understand the sustainability of the adoption and to what extent it diffused. We consider relying on community engagement and having built rapport with the participants to be a strength of the study. However, we acknowledge that the results could present a social desirability bias, as what is presented is based on perceptions and opinions of what the participants reported, and they were aware of the purpose of the study. The results of the overall study are complemented with entomological survey data.

To the best of our knowledge, there are not many studies that have applied the attributes of the innovation from DOI to the type of study we were analyzing, therefore it was challenging to define how to “measure” the attributes of the innovations proposed. However, we consider that the results presented in this manuscript provide information that can be beneficial and transferable for the implementation of integrated vector control strategies at different scales and diverse contexts.

## Conclusion

Although traditional vertical vector control approaches have had achievements, novel, and more inclusive strategies are needed to reach the goals that have not yet been met. In a 2020 report, the World Health Organization (WHO) proposed moving to an integrated approach for the control of neglected tropical diseases [35], placing people and communities as the key players in improving their health and welfare to achieve long-term sustainability and continuity of programs, which shows that the paradigm is evolving towards more horizontal approaches. In our experience, the participatory strategies applied for community engagement facilitated the adoption and sustainability of our innovation for vector and parasite reservoir control. Trialability, complexity, relative advantage, and compatibility were key to the adoption of rodent control and environmental management strategies. Future community-based interventions should be designed with these considerations to improve adoption.

### Supplementary Information

**Additional file 1.**

## Data Availability

The datasets used and/or analyzed during the current study are available from the corresponding author upon reasonable request. Data are available in aggregated form due to identifiable characteristics of participants. The IRB confidentiality agreement does not allow individual disclosure of data. A version in Spanish of this article is available in the UVG repository (link: https://repositorio.uvg.edu.gt/handle/123456789/4671).

## Abbreviations

CE: community engagement
DOI: Diffusion of Innovation Theory
ERC: Ethics Review Committee
IDRC: International Development Research Centre
KAP: knowledge, attitudes, and practices
MoH: Ministry of Health
PAR: participatory action research
PRECEDE: Predisposing, Reinforcing, and Enabling Causes in Educational Diagnosis and Evaluation
PROCEED: Policy, Regulatory and Organizational Constructs in Educational and Environmental Development
SWOT: Strengths, Weaknesses, Opportunities, Threats
TDR: Special Programme for Research and Training in Tropical Diseases
UNICEF: United Nations Children’s Fund
UNDP: United Nations Development Programme
UVG: Universidad del Valle de Guatemala
WHO: World Health Organization
