# Supplementary material for: Adoption of community-based strategies for sustainable vector control and prevention
Source: BMC Public Health. 2023 Sep 21;23:1834. doi: 10.1186/s12889-023-16516-8 (PMC10512496; doi:10.1186/s12889-023-16516-8)
Supplement: Supplementary file 1 — Additional file 1. [file 12889_2023_16516_MOESM1_ESM.pdf]

Additional File 1. Applying the Consolidated Criteria for Reporting Qualitative Studies (COREQ): 32-item Checklist to our study.

No	Item	Guide Questions	Description and/or Reference in the Article
**Domain 1: Research team and reflexivity**			
	Personal Characteristics		
1	Interviewer/ facilitator	Which author/s conducted the interview or focus group?	Elizabeth Pellecer Rivera (EPR) conducted interviews and questionnaires.<br>Margarita Rivera Arrivillaga (MRA) conducted interviews.<br>Jorge Sincal (JS) conducted questionnaires.<br>Hugo Perdomo (HP) conducted questionnaires.
2	Credentials	What were the researcher's credentials? E.g., PhD, MD	MRA and EPR had a BA degree.<br>HP had a BS degree.<br>JS is a field technician.<br>They are all Guatemalans,<br>and Spanish is their first language.
3	Occupation	What was their occupation at the time of the study?	EPR worked at UVG as a social research assistant with the project.<br>MRA had an internship at UVG as a social research assistant with the project.<br>HP worked at UVG as a research assistant.<br>JS worked at UVG as a field technician with the project
4	Gender	Was the researcher male or female?	EPR and MRA are female.<br>HP and JS are male.
5	Experience and training	What experience or training did the researcher have?	Both EPR and MRA are social<br>scientists, with studies and working experience conducting qualitative research.<br>HP is a biochemist, with studies and working experience with Chagas disease in Guatemala.<br>JS is a field technician and has experience working in the region since the project started.<br>They all fulfilled training on ethics in research with human subjects.
	Relationship with participants		
6	Relationship established	Was a relationship established prior to study commencement?	The group of researchers, led by Pamela Marie Pennington (PMP) and Sandra De Urioste-Stone (SMS), have been working in Comapa since 2009, which is when the relationship with the MoH and other institutional collaborators began.<br><br>The relationship with the participants was developed through the duration of the implementation of the study, as our team members were in Comapa on a daily basis and the visits to the communities were

7	Participant knowledge of the interviewer	What did the participants know about the researcher? e.g., personal goals, reasons for doing the research	repetitive. Especially, during the PAR process our team will visit the Intervention Group communities at least once a month for a meeting. The rapport and trust strengthen throughout the time and activities implemented.<br><br>When conducting the interviews and questionnaires, our team members would ask for verbal and written informed consent, describing the aim of the research, and solving questions that the participants may have about the study and the research team.
8	Interviewer characteristics	What characteristics were reported about the interviewer/facilitator? e.g., Bias, assumptions, reasons, and interests in the research topic	
**Domain 2: Study design**			
	Theoretical framework		
9	Methodological orientation and Theory	What methodological orientation was stated to underpin the study? e.g., grounded theory, discourse analysis, ethnography, phenomenology, content analysis	We used as a methodological framework the PRECEDE (Predisposing, Reinforcing, and Enabling Causes in Educational Diagnosis and Evaluation) - PROCEED (Policy, Regulatory and Organizational Constructs in Educational and Environmental Development) model for community interventions.<br><br>For the analysis, we used the innovation attributes proposed by the Diffusion of Innovations Theory as proposed by Rogers (1983) (Table 2).
	Participant selection		
10	Sampling	How were participants selected? e.g., purposive, convenience, consecutive, snowball	For the questionnaires, we targeted all the participants from the 9 communities in the intervention group, which were originally selected using a probability systematic sampling design.<br><br>For the interviews we used purposive sampling, using a maximum variation strategy.
11	Method of approach	How were participants approached? e.g., face-to-face, telephone, mail, email	For the questionnaires, we visited the participants' households. For the interviews, we called the selected participants or made an in-person invitation. All interviews and questionnaires were conducted face-to-face.
12	Sample size	How many participants were in the study?	208 participants for the questionnaires<br>31 participants for 25 individual interviews and 3 group interviews with 2 participants<br>18 interviews focusing on women as change agents

13	Non-participation	How many people refused to participate or dropped out? Reasons?	The intervention group consisted of 216 households, and only 208 responded to the interim evaluation questionnaire. Eight respondents were missing due to various reasons (i.e., they were busy or out of their household at the time of the visit).
	Setting		
14	Setting of data collection	Where was the data collected? e.g., home, clinic, workplace	The interviews took place in the location preferred by the participants, in their communities, mainly at the participants' houses. All the questionnaires were conducted during a house visit.
15	Presence of non-participants	Was anyone else present besides the participants and researchers?	In some cases, family members of the participants were present (i.e., children, husband), and one or more members of our research team.
16	Description of sample	What are the important characteristics of the sample? e.g., demographic data, date	As described in the Data Collection section.
	Data collection		
17	Interview guide	Were questions, prompts, guides provided by the authors? Was it pilot tested?	We developed the interview protocol, which was reviewed, and pilot tested by members of the researchers' team. The questionnaire protocol was developed based on the DOI attributes.
18	Repeat interviews	Were repeat interviews carried out? If yes, how many?	We did not conduct repeat interviews.
19	Audio/visual recording	Did the research use audio or visual recording to collect the data?	The interview audio was recorded with participant consent. Questionnaires were filled in printed documents during the house visits.
20	Field notes	Were field notes made during and/or after the interview or focus group?	Field notes were taken for interviews with the 18 women.
21	Duration	What was the duration of the interviews or focus group?	Interviews lasted between 10 – 60 minutes.
22	Data saturation	Was data saturation discussed?	We did not discuss data saturation.
23	Transcripts returned	Were transcripts returned to participants for comment and/or correction?	No, transcripts were not returned to participants.
**Domain 3: analysis and findings**			
	Data analysis		
24	Number of data coders	How many data coders coded the data?	The data generated was coded by EPR and MRA.

25	Description of the coding tree	Did authors provide a description of the coding tree?	We used five innovation attributes proposed by the Diffusion of Innovations Theory as described in Table 2 and "Data Analysis" section.
26	Derivation of themes	Were themes identified in advance or derived from the data?	
27	Software	What software, if applicable, was used to manage the data?	We used STATA17® for the quantitative analysis; and NVivo12® for the qualitative data organization and analysis.
28	Participant checking	Did participants provide feedback on the findings?	Participants did not provide feedback on these final findings.
	Reporting		
29	Quotations presented	Were participant quotations presented to illustrate the themes / findings? Was each quotation identified? *e.g., participant number*	Yes, the quotations are included in the Results section and each participant was assigned a code for confidentiality.
30	Data findings consistent	Was there consistency between the data presented and the findings?	Yes, the data presented and the findings are consistent.
31	Clarity of major themes	Were major themes clearly presented in the findings?	The major themes are presented, using five innovation attributes proposed by the Diffusion of Innovations Theory.
32	Clarity of minor themes	Is there a description of diverse cases or discussion of minor themes?	We included all the minor themes found under the major themes defined by the theoretical constructs used.
